# Supplementary material for: On the effects of alternative optima in context-specific metabolic model predictions
Source: PLoS Comput Biol. 2017 May 30;13(5):e1005568. doi: 10.1371/journal.pcbi.1005568 (PMC5469500; doi:10.1371/journal.pcbi.1005568)
Supplement: S1 Fig — A sequence of optimal solutions (i.e., flux distributions) to the leaf-specific RegrExLAD integration problem is presented. The sequence begins with λ = 0 (i.e., no regularization) and ends with λ = 1, which is the value for which all fluxes are shrunk to 0. Flux distributions get sparser with increasing values for lambda. In addition, the total entropy of the alternative optima tends to decrease with increasing values for lambda. This indicates the existence of a trade-off between sparsity and entropy reduction. In this study, a mild regularization (λ = 0.1) seems sufficient to substantially reduce the total entropy value while preventing flux distributions to become too sparse (i.e., in which important reactions for a given context may be excluded). (PDF) [file pcbi.1005568.s001.pdf]

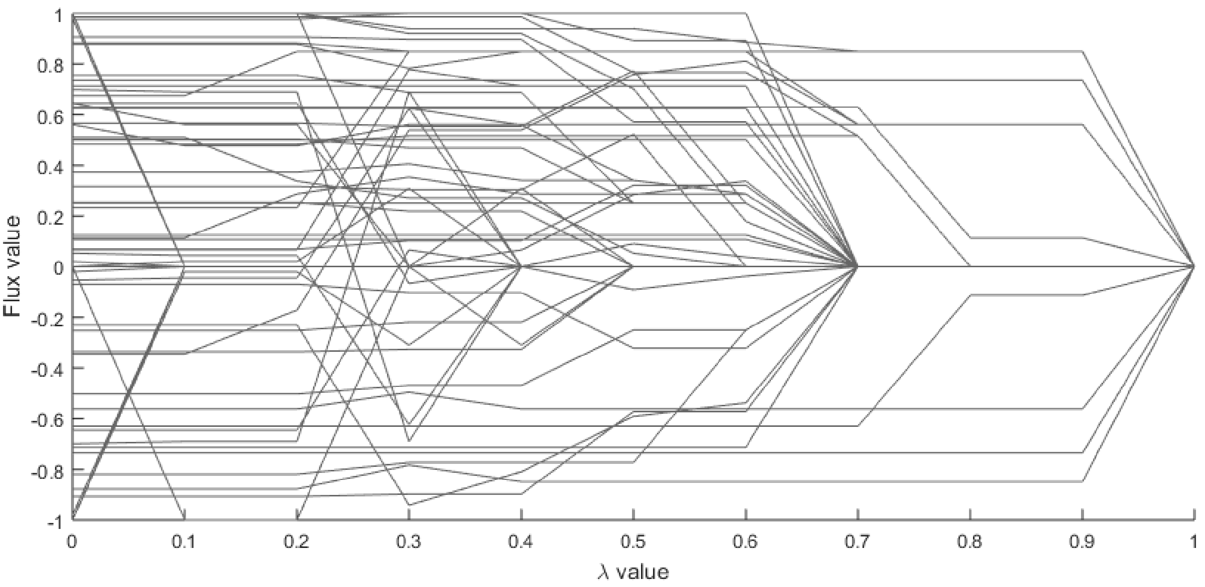

S1 Fig. The RegrEx$_{LAD}$ solution path through a sequence of increasing λ-values. A sequence of optimal solutions (*i.e.*, flux distributions) to the leaf-specific RegrEx$_{LAD}$ integration problem is presented. The sequence begins with λ = 0 (*i.e.,* no regularization) and ends with λ = 1, which is the value for which all fluxes are shrunk to 0. Flux distributions get sparser with increasing values for lambda. In addition, the total entropy of the alternative optima tends to decrease with increasing values for lambda. This indicates the existence of a trade-off between sparsity and entropy reduction. In this study, a mild regularization (λ = 0.1) seems sufficient to substantially reduce the total entropy value while preventing flux distributions to become too sparse (*i.e.*, in which important reactions for a given context may be excluded).
