# Supplementary material for: On the effects of alternative optima in context-specific metabolic model predictions
Source: PLoS Comput Biol. 2017 May 30;13(5):e1005568. doi: 10.1371/journal.pcbi.1005568 (PMC5469500; doi:10.1371/journal.pcbi.1005568)
Supplement: S1 Appendix — This appendix compares the original formulation of RegrEx (RegrExOLS) with the formulation used in this study (RegrExLAD) and justifies its usage. A comparison of the performance between the two versions is also provided. (PDF) [file pcbi.1005568.s011.pdf]

S1 Appendix. Detailed description of RegrEx$_{\mathrm{LAD}}$ and comparison with the original RegrEx$_{\mathrm{OLS}}$

The RegrEx$_{LAD}$ method

In this appendix we justify the usage of RegrEx$_{\mathrm{LAD}}$ in this study, instead of the original RegrEx version presented in [1]. As commented in the main text, the original RegrEx method minimizes the squared ℓ_2 norm of $\epsilon = d - v$, the difference vector between the experimental data vector, d, and a feasible flux distribution, v. This is indeed the only difference with respect to the RegrEx$_{\mathrm{LAD}}$ method, which minimizes the ℓ_1 norm (*i.e.*, the sum of absolute error values) of ϵ. To this end, RegrEx solves the mixed integer quadratic program:

$$
\begin{aligned}
& v_{opt} = \underset{\substack{\epsilon=[\epsilon_{irr};\epsilon_{for};\epsilon_{rev}]\in\mathbb{R} \\ v=[v_{irr};v_{for};v_{rev}]\in\mathbb{R}_0^+, \\ x\in\{0,1\}^n}}{\arg\min} \tfrac{1}{2}\|\epsilon\|_2^2 + \lambda \| v \|_1 \\
& s.t. \\
& 1.\ S_{ext} v = 0 \\
& \left.\begin{aligned}
& 2.\ v_{irr_i} + \epsilon_{irr} = d_{irr} \\
& 3.\ v_{for_i} + \epsilon_{for} - x_i d_{revRxns} = d_{revRxns} \\
& 4.\ v_{rev_i} + \epsilon_{rev} - x_i d_{revRxns} = 0
\end{aligned}\right\}, \quad i \in R_D \qquad \text{(OP}_1\text{)}, \\
& 5.\ v_{irr\min} \le v_{irr} \le v_{irr\max} \\
& 6.\ v_{for} + x v_{for\min} \ge v_{for\min} \\
& 7.\ v_{rev} - x v_{rev\min} \ge 0 \\
& 8.\ v_{for} + x v_{for\max} \le v_{for\max} \\
& 9.\ v_{rev} - x v_{rev\max} \le 0
\end{aligned}
$$

as opposed to the mixed integer linear program solved by RegrEx$_{\mathrm{LAD}}$ (presented in the main text):

$$
\begin{aligned}
& v_{opt} = \underset{\substack{\epsilon^+=[\epsilon^+_{irr};\epsilon^+_{for};\epsilon^+_{rev}], \\ \epsilon^-=[\epsilon^-_{irr};\epsilon^-_{for};\epsilon^-_{rev}], \\ v=[v_{irr};v_{for};v_{rev}]\in\mathbb{R}_0^+, \\ x\in\{0,1\}^n}}{\arg\min} (\epsilon^+ + \epsilon^-) + \lambda \| v \|_1 \\
& s.t. \\
& 1.\ S_{ext} v = 0 \\
& \left.\begin{aligned}
& 2.\ v_{irr_i} + (\epsilon^+_{irr} - \epsilon^-_{irr}) = d_{irr} \\
& 3.\ v_{for_i} + (\epsilon^+_{for} - \epsilon^-_{for}) + x d_{revRxns} = d_{revRxns} \\
& 4.\ v_{rev_i} + (\epsilon^+_{rev} - \epsilon^-_{rev}) - x d_{revRxns} = 0
\end{aligned}\right\}, \quad i \in R_D \qquad \text{(OP}_2\text{)} \\
& 5.\ v_{irr\min} \le v_{irr} \le v_{irr\max} \\
& 6.\ v_{for} + x v_{for\min} \ge v_{for\min} \\
& 7.\ v_{rev} - x v_{rev\min} \ge 0 \\
& 8.\ v_{for} + x v_{for\max} \le v_{for\max} \\
& 9.\ v_{rev} - x v_{rev\max} \le 0
\end{aligned}
$$

During the course of this study, we first tried to investigate the alternative optima space of RegrEx through a sampling procedure, in a way akin to the Variability Flux Sampling procedure implemented in [2]. The Variability Flux Sampling procedure was developed to investigate the alternative optima space of the InGenMinimizer method (presented in the same publication), by

generating a random sample of alternative optimal flux distributions. The InGenMinimizer method follows the quadratic program,

$$
\begin{aligned}
& Z_{opt} = \min_{v,\varepsilon} \tfrac{1}{2} \| \varepsilon \|_2^2 \\
& s.t. \\
& 1.\ Sv = 0 \qquad\qquad\qquad (\text{OP}_3). \\
& 2.\ v_{\min} \le v \le v_{\max} \\
& 3.\ v_i = d_i + \varepsilon_i, \forall i \in R_D
\end{aligned}
$$

Therefore RegrEx (OP_1) can be seen as an extension of the InGenMinimizer method (OP_3), in which (*i*) $\ell 1$-regularization is included in the objective function, and (*ii*) reversible reactions with associated data are also taken into account in the minimization, which requires introducing a vector of binary variables, x (as explained in the main text).

The Variability Flux Sampling procedure was formulated as the quadratic program,

$$
\begin{aligned}
& \min_{v,\varepsilon,\delta} \tfrac{1}{2} \| \delta \|_2^2 \\
& s.t. \\
& 1.\ Sv = 0 \\
& 2.\ v_{\min} \le v \le v_{\max} \qquad\qquad (\text{OP}_4), \\
& \left.\begin{aligned} & 3.\ v_i = d_i + \varepsilon_i \\ & 4.\ \tfrac{1}{2} \| \varepsilon \|_2^2 = Z_{opt} \end{aligned}\right\}, \forall i \in R_D \\
& 5.\ v = v_{rand} + \delta
\end{aligned}
$$

which minimizes the distance, δ, between an alternative optimal flux distribution, v, and a randomly generated flux distribution, v_{rand} (OP_4 is solved *n* times to obtain a sample of *n* alternative optimal flux distributions). The key in OP_4 is constraint number 4, *i.e.*, $\frac{1}{2}\|\epsilon\|_2^2 = Z_{opt}$, which guarantees that any sampled feasible v, is also optimal, since it renders the same squared ℓ_2 norm of ϵ previously obtained by OP_3. This is a quadratic equality constraint, which makes OP_4 non-convex and thus intractable by convex optimization tools. Note that this constrain would also be required in the case of RegrEx, since it also minimizes the squared ℓ_2 norm of ϵ.

In the Variability Flux Sampling procedure, authors used a non-convex solver, MINOS [3], to tackle this problem. However, several aspects make the case of RegrEx more complex: firstly, in the Variability Flux Sampling procedure authors only dealt with seven reactions with associated data, in contrast, RegrEx must evaluate all reactions with associated data in a GEM. Secondly, integer variables were not required in the Variability Flux Sampling procedure, since all seven reactions were irreversible, as oppose to RegrEx, where reversible reactions with associated data are also taken into account. Lastly, a flux distribution that is alternatively optimal to RegrEx must also render the same ℓ_1 norm as the original optimum, thus a second constraint like $\|v_s\|_1 = \|v_{opt}\|_1$ has to be added. Altogether, these particularities make the optimization problem associated to any RegrEx alternative optima sampling procedure hardy tractable by any existing solver. However, it is computationally tractable to sample alternative optimal solutions of $\text{RegrEx}_{\text{LAD}}$. This is because the objective function of $\text{RegrEx}_{\text{LAD}}$ is linear, and hence only two linear constraints are required to guarantee that a sampled

flux distribution is optimal to $RegrEx_{LAD}$. Thus the sampling procedure ($RegrEx_{AOS}$, see main text) can be casted as a convex optimization problem and solved with existing solvers.

Although computational tractability was our main motivation to develop $RegrEx_{LAD}$, we noted that this alternative version may have another advantage over the original (renamed $RegrEx_{OLS}$ in the following). $RegrEx_{OLS}$ and $RegrEx_{LAD}$ parallel two classical approaches followed in linear regression, namely, the ordinary least squares (OLS) and the least absolute deviations (LAD, also known as least absolute value, LAV) method [4]. OLS and LAD regression behave differently upon the presence of outliers in the distribution of errors (*i.e.*, the vector ϵ), that is, elements that are very far away from the mean of the distribution. Concretely, the OLS method tends to get biased results in such cases, since the squared ℓ_2 norm of ϵ gives excessive importance to these elements. On the other hand, the LAD method is more robust under the presence of such outliers, and thus less prone to give biased results (in fact, the LAD method is the simplest among the so-called Robust Regression techniques, see for instance [5]). In the context of RegrEx, this means that $RegrEx_{LAD}$ could be a better choice in cases where outliers are present in the error distribution, for instance, if a given mapped gene expression value is particularly high with respect to the mean value of the gene expression data set. In fact, this idea has been implemented in the case of the least absolute shrinkage and selection (LASSO) operator [6] (which inspired the development of $RegrEx_{OLS}$), which applies a ℓ_1-regularization to an OLS regression. Concretely, the LASSO has been adapted to a LAD regression, showing advantages in cases where the distribution of errors is not appropriate for OLS estimation [7].

To test the previous idea, we evaluated the $RegrEx_{OLS}$ and $RegrEx_{LAD}$ performance under the inclusion of outliers in the leaf data set used in the main study. To this end, we first generated a sample of randomly perturbed leaf data vectors, $d^*_{Leaf(j)} = d_{Leaf} + \mu_{(j)}$, $j = \{1, \dots, 10^4\}$, obtained by adding a uniform noise ,$\mu_{(j)}$, (±1% of the mean value of d_{Leaf}) to the original leaf data set, d_{Leaf}. We next obtained a "contaminated" leaf data set, which contained an outlying expression value for one of the reactions. Concretely, we substituted the data associated to the reaction that had the minimum value in d_{Leaf} by a large amount, in this case 5 times the maximum value in d_{Leaf}. We then applied $RegrEx_{OLS}$ and $RegrEx_{LAD}$ using the AraCOREred model and the contaminated leaf data set, and calculated the total sum of the absolute errors,

$$T_{\epsilon_{(j)}} = \frac{1}{R_D} \sum_j v_{(i,j)} - d^*_{Leaf(i,j)} \,,$$

with $i = \{1, \dots, R_D\}$, where R_D corresponds to the number of reactions with associated data, between the optimum $RegrEx_{OLS}$ and $RegrEx_{LAD}$ flux distributions and each of the perturbed leaf datasets, $d^*_{Leaf(j)}$, in the sample (the code used in this evaluation is included in SFile1). In this evaluation, $RegrEx_{LAD}$ rendered smaller total sums of absolute errors across the perturbed data sample (mean $T_\epsilon = 0.718$ in $RegrEx_{LAD}$ *versus* a mean $T_\epsilon = 0.722$ in $RegrEx_{OLS}$) as determined by a two-sided Mann-Whitney test (p-value = 0). In addition, $RegrEx_{LAD}$ did not render smaller total sums when the original ("uncontaminated") leaf data set was used under the same setting (mean $T_\epsilon = 0.709$ in $RegrEx_{LAD}$ *versus* a mean $T_\epsilon = 0.706$ in $RegrEx_{OLS}$, p-value = 1) . Although the reported differences between total sums of absolute errors are small, they serve to illustrate the more robust behavior of $RegrEx_{LAD}$ under the presence of outliers.

References

1. Robaina Estévez S, Nikoloski Z. Context-Specific Metabolic Model Extraction Based on Regularized Least Squares Optimization. PLoS One. Public Library of Science; 2015;10:

e0131875. doi:10.1371/journal.pone.0131875

2. Recht L, Töpfer N, Batushansky A, Sikron N, Gibon Y, Fait A, et al. Metabolite Profiling and Integrative Modeling Reveal Metabolic Constraints for Carbon Partitioning under Nitrogen-Starvation in the Green Alga Haematococcus pluvialis. J Biol Chem. 2014;289: 30387–30403. doi:10.1074/jbc.M114.555144

3. Bruce A. Murtagh MAS. Minos User’s Manual. Syst Optim Lab Dep Oper Res Stanford Univ.

4. Lawrence KD, Shier DR. A comparison of least squares and least absolute deviation regression models for estimating Weibull parameters. Commun Stat - Simul Comput. Taylor & Francis Group; 2010; Available: http://www.tandfonline.com/doi/abs/10.1080/03610919808813515a

5. Dielman TE. Least absolute value regression: recent contributions. J Stat Comput Simul. Taylor & Francis; 2005;75: 263–286. doi:10.1080/0094965042000223680

6. Tibshirani R. Regression Selection and Shrinkage via the Lasso. J R Stat Soc B. 1994;58: 267–288. doi:10.2307/2346178

7. Wang H, Li G, Jiang G. Robust Regression Shrinkage and Consistent Variable Selection Through the LAD-Lasso. Journal of Business & Economic Statistics. 2007. pp. 347–355. doi:10.1198/073500106000000251
