# Supplementary material for: On the effects of alternative optima in context-specific metabolic model predictions
Source: PLoS Comput Biol. 2017 May 30;13(5):e1005568. doi: 10.1371/journal.pcbi.1005568 (PMC5469500; doi:10.1371/journal.pcbi.1005568)
Supplement: S2 Appendix — This appendix describes the alternative procedure (iMATAOS) to sample the alternative optima space of iMAT, which is proposed in this study. We apply iMATAOS to evaluate the ambiguity of the predictions in both, the leaf and the liver scenarios. (PDF) [file pcbi.1005568.s012.pdf]

S2 Appendix. Description of $iMAT_{AOS}$ and application to the two evaluated case studies

Alternative optimal solutions in the iMAT method: background

As mentioned in the main text, there already exists a procedure to investigate the alternative optima space for iMAT [1]. Hence, we considered relevant to apply iMAT to the same context-specific reconstructions examples used in CorEx, and analyze its alternative optimal solutions. Here, we briefly summarize the iMAT method as well as the procedure proposed by the authors to analyze its alternative optima. In addition, we present our novel complementary approach to sample the alternative optima space of iMAT. iMAT aims at maximizing the global similarity between a given expression data set and a feasible flux distribution of the GEM where data is being integrated. Therefore, in this sense, it follows an approach similar to RegrEx. However, iMAT does not directly minimize the distance between data and flux values. Instead, iMAT first integrates experimental information by classifying reactions in the GEM into two groups: one, R_H, populated by reactions with *highly* expressed associated genes (*i.e.*, above a fixed threshold value, ϵ) and another, R_L, by reactions with *lowly* expressed associated genes (*i.e.*, below ϵ). The MILP presented in OP_5 is then solved to maximize the number of active reactions in R_H (with non-zero flux) and the number of inactive reactions in R_L (with zero flux value), subject to the usual mass balance and thermodynamic constraints. This is implemented by maximizing the norm of two vectors of binary variables, y^+, y^-, that select reactions in R_H to be active and reactions in R_L to be inactive (the extra variable, y^-, is added to account for reversible reactions).

$$Z_{opt} = \max_{\substack{v \in \mathbb{R}^m \\ y^+, y^- \in \{0,1\}}} \sum_{i \in R_H} (y_i^+ + y_i^-) + \sum_{i \in R_L} y_i^+$$

$$\begin{aligned}
&s.t.\\
&1.\ Sv = 0\\
&2.\ v_{\min} \le v \le v_{\max} \qquad\qquad (OP_5)\\
&3.\ v_i + y_i^+ (v_{\min,i} - \varepsilon) \ge v_{\min,i}, i \in R_H\\
&4.\ v_i + y_i^- (v_{\max,i} + \varepsilon) \le v_{\max,i}, i \in R_H\\
&5.\ v_{\min,i}(1 - y_i^+) \le v_i \le v_{\max,i}(1 - y_i^+), i \in R_L
\end{aligned}$$

To deal with alternative optimal flux distributions, authors [1] proposed the following approach, which we denominate here $iMAT_{FVA}$. First, OP_5 is solved twice for each reaction in the GEM; the first time, the reaction is forced to be active, in the second, to be inactive. The two objective values, $Z_{act(i)}$, $Z_{inac(i)}$, corresponding to the optimizations where reaction i was active and inactive, respectively, are then compared. If $Z_{act(i)} > Z_{inac(i)}$, reaction i is considered to be active (with confidence $Z_{act(i)} - Z_{inac(i)}$), if $Z_{act(i)} < Z_{inac(i)}$, is considered to be inactive (with confidence $Z_{act(i)} - Z_{inac(i)}$) and if $Z_{act(i)} = Z_{inac(i)}$ is taken as undetermined under the data set been integrated. Therefore, $iMAT_{FVA}$ determines the sets of reactions that individually increase the global similarity to data when active and inactive, respectively, and the set of reactions that do not affect the optimum global similarity to data under whatever state, active or inactive. However, it does not provide information about how the states of reactions distribute across the alternative optima space of iMAT. For instance, a given reaction could be

classified as active and still be either active or inactive across the space of all alternative optimal flux distributions generated by iMAT.

We emphasize that the results obtained through iMAT_{FVA} do not align qualitatively to the ones obtained for RegrEx and CorEx (by extension FastCORE and CORDA), and hence they have to be interpreted on their own. To make a fair comparison, we need a method that allows drawing samples of alternative optimal flux distributions to iMAT. In the EXAMO publication [2], authors generated such a sample by collecting the flux distributions that rendered the maximum objective value (Z_{opt} in OP_5) when applying iMAT_{FVA}. Therefore, we can only generate a limited number of sampled optimal flux distributions with this method. Here, we propose a different procedure to evaluate the alternative optimal space of iMAT (iMAT_{AOS}), which follows a similar approach to the one employed by $\text{RegrEx}_{\text{AOS}}$: we first generate a random flux distribution, v_{rand}, and then search for the closest feasible flux distribution, v, that renders the same optimal result, Z_{opt}, found by iMAT. iMAT_{AOS} optimizes the mixed integer quadratic program:

$$
\begin{aligned}
&\min_{\substack{v,\delta\in\mathbb{R}^m \\ y^+,y^-\in\{0,1\}}} \ \tfrac{1}{2}\|\delta\|_2^2 \\
&s.t. \\
&1-5 \quad (\text{OP}_5) \qquad\qquad\qquad\qquad (\text{OP}_6). \\
&6.\ v = v_{rand} + \delta \\
&7.\ \textstyle\sum_{i\in R_H}(y_i^+ + y_i^-) + \sum_{i\in R_L} y_i^+ = Z_{opt}
\end{aligned}
$$

OP_6 inherits constraints 1-5 from OP_5 and includes constraint 6, which defines the distance, $\delta = v - v_{rand}$ to be minimized, and constraint 7, which guarantees that v remains within the alternative optimal space of the previous iMAT optimization. In this manner, iMAT_{AOS} allows drawing an unlimited number of random alternative flux distributions that are optimal to OP_5.

Alternative optimal solutions in the iMAT method: case studies

We next applied iMAT and iMAT_{FVA}—to analyze its alternative optimal solutions—to AraCOREred and Recon1red. In this case, we used the core set of reactions for the leaf and the liver contexts as the R_H group in iMAT. In this way, we obtained a leaf-specific model containing 131 reactions and 154 metabolites, while the liver-specific model consisted of 1235 reactions and 1067 metabolites. By applying iMAT_{FVA}, we found a total of 272 active, 178 inactive and 5 undetermined reactions across the iMAT alternative optima space for the leaf context. For the liver context, the alternative optima space included 1223 active, 981 inactive and 143 undetermined reactions in the case of liver (Table 3). We quantified the uncertainty of the iMAT data integration problem by taking the proportion of undetermined reactions over the total number in the GEM. The undetermined reactions in the alternative optima space for the leaf and the liver- contexts were 1.1% and 6.1%, respectively.

	Leaf				Liver			
	**A**	**I**	**U**	$\overline{M_R}$ **(CV)**	**A**	**I**	**U**	$\overline{M_R}$ **(CV)**
**iMAT$_{FVA}$**	272	178	5	-	1223	981	143	-
**iMAT$_{AOS}$**	275	40	140	43.16(0.20)	1069	247	1153	369.32(0.05)
**Overlap**	259 (95.2%)	35 (19.7%)	0	-	928 (75.9%)	69 (5.6%)	2 (0.16%)	-

Table 3. Summary of the alternative optima space of iMAT. This table includes the number of active, A, inactive, I, and undetermined, U, reactions across the alternative optima space as determined by iMAT$_{FVA}$ and the iMAT$_{AOS}$. The intersection between the two methods is also displayed for each of the three categories (Overlap). Finally, the mean number of reaction mismatches (*i.e.*, the Hamming distance), $\overline{M_R}$, between the generated alternative optimal networks (see main text) is also displayed (the coefficient of variation, CV, is shown in parenthesis). These figures are displayed for the leaf- and the liver-specific scenario.

We next evaluated the alternative optimal space with iMAT$_{AOS}$, which allowed us to draw two random samples (size $n = 2000$) of leaf- and liver-specific alternative optimal flux distributions. We focused on characterizing the state of the reactions, as active or inactive, across the sample. For the leaf context, 60% of the reactions were active in all alternative flux distributions, 9.23% had a fixed inactive state, and a 30.8% were of undetermined state across the sample. For the liver context, the fraction of fixed active reactions amounted to a 43.3%, 9.52% showed fixed inactive state, and 47.2% of the reactions were of undetermined state across the sample (Table 3). Here, too, we considered the fraction of reactions with undetermined state across the alternative optima sample as an uncertainty measure of the iMAT data integration problem. Our results demonstrated that the uncertainty for the liver context was greater than that for the leaf (Table 3), which agrees with the results previously obtained in the case of CorEx. These findings were supported by the significantly different Hamming distance calculated between any possible pair of alternative optimal networks (one-sided ranksum test, p-value = 0, see Methods).

Additionally, a comparison of the results obtained through the two alternative methods, iMAT$_{FVA}$ and iMAT$_{AOS}$, showed a good agreement in the sets of reactions classified as active across the alternative optima space: a 94.8% of active reactions per iMAT$_{FVA}$ were also found active by iMAT$_{AOS}$ in the leaf context, and a 75.9 % for the liver context. However, this agreement did not hold in the case of inactive and undetermined reactions, both in leaf and in liver (Table 3). Therefore, this comparison highlighted the importance of analyzing a sample of alternative optimal solutions to obtain a more complete understanding of the uncertainty associated to an experimental data integration problem.

iMAT implementation and alternative optima evaluation

The iMAT implementation was taken from the function *createTissueSpecificModel* in the COBRA toolbox [3] (for MATLAB) and slightly modified to allow the usage of the Gurobi solver (version 7.01), used throughout this study. In addition, the iMAT$_{FVA}$ procedure was performed through adapting the previous iMAT implementation (no publicly available implementation of this procedure was found). Both MATLAB functions can be found in S1File under the names of *iMAT* and *iMAT$_{FVA}$*. In addition, the implementation of our alternative sampling method, *iMAT$_{AOS}$*, can be found in the same file.

References

1. Shlomi, T., Cabili, M. N., Herrgård, M. J., Palsson, B. Ø. & Ruppin, E. Network-based prediction of human tissue-specific metabolism. *Nat. Biotechnol.* **26,** 1003–1010 (2008).

2. Rossell, S., Huynen, M. A. & Notebaart, R. A. Inferring Metabolic States in Uncharacterized Environments Using Gene-Expression Measurements. *PLoS Comput. Biol.* **9,** (2013).

3. Hyduke, D. *et al.* COBRA Toolbox 2.0. *Protocol Exchange* (2011). doi:10.1038/protex.2011.234
